# Field Epidemiology Training Program Response to COVID-19 During a Conflict: Experience From Yemen

**DOI:** 10.3389/fpubh.2021.688119

**Published:** 2021-11-22

**Authors:** Abdulwahed Abduljabar Al Serouri, Yasser Ahmed Ghaleb, Labiba Anam Al Aghbari, Mohammad Abdullah Al Amad, Abdulhakem Sharaf Alkohlani, Khaled Abdullah Almoayed, Aisha Obad Jumaan

**Affiliations:** ^1^Field Epidemiology Training Program, Ministry of Public Health and Population, Sana'a, Yemen; ^2^General Directorate for Disease Surveillance and Control, Ministry of Public Health and Population, Sana'a, Yemen; ^3^Coordinator of Health Projects, Mercer Island, WA, United States

**Keywords:** COVID-19, epidemic response, conflict, health workforce, field epidemiology training program, Yemen

## Abstract

COVID-19 pandemic has underscored the need for a well-trained public health workforce to save lives through timely outbreaks detection and response. In Yemen, a country that is entering its seventh year of a protracted war, the ongoing conflict severely limited the country's capacity to implement effective preparedness and response measures to outbreaks including COVID-19. There are growing concerns that the virus may be circulating within communities undetected and unmitigated especially as underreporting continues in some areas of the country due to a lack of testing facilities, delays in seeking treatment, stigma, difficulty accessing treatment centers, the perceived risks of seeking care or for political issues. The Yemen Field Epidemiology Training Program (FETP) was launched in 2011 to address the shortage of a skilled public health workforce, with the objective of strengthening capacity in field epidemiology. Thus, events of public health importance can be detected and investigated in a timely and effective manner. During the COVID-19 pandemic, the Yemen FETP's response has been instrumental through participating in country-level coordination, planning, monitoring, and developing guidelines/standard operating procedures and strengthening surveillance capacities, outbreak investigations, contact tracing, case management, infection prevention, and control, risk communication, and research. As the third wave is circulating with a steeper upward curve than the previous ones with possible new variants, the country will not be able to deal with a surge of cases as secondary care is extremely crippled. Since COVID-19 prevention and control are the only option available to reduce its grave impact on morbidity and mortality, health partners should support the Yemen FETP to strengthen the health system's response to future epidemics. One important lesson learned from the COVID-19 pandemic, especially in the Yemen context and applicable to developing and war-torn countries, is that access to outside experts becomes limited, therefore, it is crucial to invest in building national expertise to provide timely, cost-effective, and sustainable services that are culturally appropriate. It is also essential to build such expertise at the governorate and district levels, as they are normally the first respondents, and to provide them with the necessary tools for immediate response in order to overcome the disastrous delays.

## Introduction

Six years into a war that has killed over 233,000 people, Yemen remains the largest humanitarian crisis in the world (1). The targeting of infrastructures, blockades of major airports and ports, and restrictions and diversions of humanitarian supplies have intensified conflict-induced consequences especially on health and nutrition. The country is currently experiencing the world's worst food security crisis with 24 million of the 30 million population in need of humanitarian assistance in 2020, and 1.2 million pregnant or breastfeeding women and 2.3 million children under 5 requiring treatment for acute malnutrition (2, 3).

The conflict has also gravely affected the already debilitated health system and made access to health services extremely challenging, as around 49% of health facilities are either partially functioning or non-functioning due to damage, shortages of staff, medicines, fuel, and other critical supplies (4, 5). The country is currently facing exceptionally high rates of communicable and non-communicable diseases and poor delivery of basic health services, especially preventive services such immunization. Moreover, as Yemen has been under blockade for the last 6 years, medical care as well as laboratories capacity to investigate/confirm and deal with outbreaks have been severely compromised by the shortages of medical supplies and reagents that diminish preparedness and response abilities of the health system. The situation is further complicated by the presence of high numbers of migrants, refugees and internally displaced people (6–8).

The ongoing war, political instability and fragmentation of the health system have increased the risk of large outbreaks such as cholera and diphtheria (9–11). The situation is exacerbated by the shortage of skilled public health workers, especially epidemiologists posing challenges for building effective public health systems capable of preparing and responding to the public health emergencies and the current upsurge of outbreaks (12, 13).

### COVID-19 Epidemic in Yemen

The country's first confirmed COVID-19 case was recorded on April 10, 2020 in Hadhramaut governorate and by 29 March 2021, the WHO reported 4,037 confirmed cases of COVID-19 with 852 deaths (14).

The current state of conflict and the fragile healthcare system hindered the implementation of the necessary International Health Regulations (IHR) measures and limited the capacity to adopt and implement effective preparedness and response measures to the COVID-19 epidemic. This made it difficult to trace cases and hard to implement control measures (15–18).

The COVID-19 pandemic has further underlined Yemen's public health vulnerabilities and further burdened the already overwhelmed healthcare system. Only half of health facilities are currently functional; however, they are unprepared and poorly equipped to cope with the COVID-19 epidemic. The majority do not have isolation rooms, Intensive Care Units (ICU) beds, essential medical supplies, or protective equipment for personnel (19–22). Furthermore, the testing for COVID-19 proved to be challenging both logistically and financially making early and large-scale diagnosis of COVID-19 impossible (23).

The above-mentioned vulnerabilities and constraints manifested in a misleading low official coronavirus caseload, the lowest in the Middle East and raised concerns that the virus could be circulating within communities undetected and unmitigated (24, 25). Thus, it was challenging to deliver accurate and actionable information to potentially affected populations (26). Furthermore, the 28% COVID-19 fatality rate in Yemen is among the highest in the world at five times the global average (25, 27–29). Finally, the COVID-19 epidemic is disproportionately damaging one of Yemen's most critical human resources where only 3 months after reporting the first case, 97 COVID-19 deaths were reported among health workers (30).

### The Yemen Field Epidemiology Training Program

In 2011, the Ministry of Public Health and Population (MoPHP) supported by the USA Center for Disease Control and Prevention (CDC), Atlanta, Georgia, launched the Yemen Field Epidemiology Training Program (Yemen FETP). The aim is to address pressing public health challenges arising from an increasing burden of communicable and non-communicable diseases due to a critical shortage of human resources. The Yemen FETP gained later on support from the World Health Organization (WHO), the Training Program in Epidemiology and Public Health Interventions Network (TEPHINET), and the Eastern Mediterranean Public Health Network (EMPHNET). The main goal of the Yemen FETP is to build sustainable capacity within the MoPHP to detect, investigate and respond to outbreaks; analyze and evaluate disease surveillance systems; use population-based health data to estimate the burden of injuries and communicable and non-communicable diseases; evaluate the impact of health-related interventions; and use data for policy-making and decision-making (13).

Based on a philosophy of “learning while doing,” the Yemen FETP has two training programs (i) a 2 year Advance Epidemiology Program and (ii) 3 months Basic Field Epidemiology (BFE)/Public Health Empowerment Program (PHEP).

The Yemen FETP has strengthened the capacity of the Yemen health workforce and has been instrumental in supporting the MoPHP during outbreaks and health emergencies especially during the current crisis as the security situation deteriorated and access to outside experts became limited (13). The Program has proved to be an important tool for strengthening the national public health preparedness and response. Through focusing on timely investigation, adherence to standard case definition, proper case management, effective infection control practice and strengthening surveillance system, the Yemen FETP participated effectively in many outbreak's responses including the world's worst cholera epidemic that started in 2016 (31). Studies from other developing countries showed that FETPs play a critical role in building human resource capacity, strengthening public health systems, and fighting against epidemic (32–37).

### Response of the Yemen FETP to COVID-19 Epidemic

COVID-19 has underscored the need for a well-trained public health workforce and the importance of the FETPs to expand and reinforce the epidemiologic capacity to ensure the timely detection and response to outbreaks and save lives (38–41).

We used secondary data available at the Yemen FETP e.g., training/activities performed, monthly, quarterly, and outbreak investigation reports that are relevant to the WHO nine pillars of COVID-19 preparedness and response to document the Yemen FETP response to COVID-19. These pillars were developed by WHO and updated on 22 May 2020 to provide a practical guide that may be used by national authorities to develop and update their COVID-19 national preparedness and response plans. It is also intended for use by key partners to develop or update their COVID-19 multiagency plans with and in support of national authorities (42).

### Pillar 1: Country-Level Coordination, Planning, and Monitoring

Immediately after the COVID-19 was declared by the WHO as a public health emergency of international concern (PHEIC) on January 30, 2020, the MoPHP formed the National Technical Coordinating Committee (NTCC) to activate the multi-sectoral and multi-partner coordination mechanisms to support preparedness and response. The Yemen FETP participated in the NTCC high-level coordination meetings where it provided technical advice on the coordinated management of COVID-19. Furthermore, the Yemen FETP conducted an assessment for the risk of COVID-19 importation to Yemen which revealed that the Yemen has a moderate to high risk. Accordingly, four scenarios were developed to deal with the epidemic that was approved by the NTCC and the High Outbreaks Committee.

Yemen FETP staff and graduates also led the COVID-19 risk assessments using the WHO National capacities review tool for a novel coronavirus (nCoV) to better understand existing capacities in areas of detection and response (43). Thereafter, they participated in a simulation exercise to assess the country's readiness to face any possible COVID-19 epidemic where operational plans with estimated resource requirements were developed with national authorities and key partners.

Furthermore, the Yemen FETP used the Imperial College Model to identify the most likely scenarios with mitigated or unmitigated strategies around COVID-19 transmission to estimate the total numbers infected, deaths, hospital and critical bed demands at the national and governorate levels (44).

### Pillar 2: Risk Communication and Community Engagement

The Yemen FETP Director was the spokesman for the NTCC at the beginning of the epidemic and provided the public with regular messages on the development of the pandemic. Yemen FETP also supported the development of COVID-19 local educational materials, adapted a wide range of other material to the Yemen context and shared them with the National Centre for Health Education. The program also reviewed and provided feedback on the printed educational materials before its wider distribution.

In March 2020, the MoPHP supported by WHO established COVID-19 telephone hotlines that was supervised by the Yemen FETP which developed the hotlines objectives and the guidelines on best practices for public health emergency. The Yemen FETP graduates managed the hotline 24 h a day, 7 days a week; received reports on suspected COVID-19 cases and coordinated with the responsible rapid response teams for investigation. They also responded to questions, provided accurate and timely information, and addressed the common misconceptions and rumors. From March 2020 to December 2020, the hotline received 10,516 calls including 646 (25%) calls that reported a suspected case; of these 65 (2%) suspected deaths. About 304 (11%) callers were advised to go to hospitals and 337 (13%) were investigated by the RRT (See Table 1).

**Table 1:** COVID-19 hotline data analysis, Yemen, March–December 2020.

**Number of calls by month (*n* = 10,516)**	**No**.	**%**
April	3,001	28
May	2,471	23
June	1,894	18
July	904	9
August	482	4
September	594	6
October	481	5
November	413	4
December	276	3
**Type of call (*****n*** **=** **10,545)**		
Queries/information	7,710	73
Reporting suspected case	2,646	25
Rumors	189	2
**Gender of callers (*****n*** **=** **10,356)**		
Male	8,870	86
Females	1,486	14
**Governorate (*****n*** **=** **10,356)**		
Sana'a City	3,509	34
Sana'a Governorate	950	9
Taiz	937	9
Ibb	742	7
Dhamar	633	6
Amran	577	6
Sadda'h	520	5
Haja'h	419	4
Al Hodeida	373	4
Al Bayda	254	2
Other Southern and Eastern governorates	1,452	14
**Reported symptoms (*****n*** **=** 2,646)^*^		
Fever	1,800	68
Cough	1,272	48
Sore throat	671	25
Headache	535	20
Runny Nose	531	20
Shortness of breath	372	14
Other	1,406	53
**Reported deaths (*****n*** **=** **2,646)**	65	2
**Type of inquiry (*****n*** **=** **7,710)**		
Symptoms Inquiries	4,160	54
Mode of transmission	1,708	22
Prevention measures	1,842	24
**Advice /referral (*****n*** **=** **2,620)**		
Symptomatic Treatment/preventive measures	1,672	64
RRT	337	13
Hospital	304	11
Home isolation	313	12

* *Multiple answers allowed*.

Finally, the Yemen FETP added a special section in its website (http://www.yfetp.com) and launched a special WhatsApp group where Arabic/English technical materials and updates on the COVID-19 were posted. The WhatsApp group was used as a forum for discussion, sharing opinions, asking questions and getting scientific guidance.

### Pillar 3: Surveillance, Rapid Response Teams, and Case Investigation

One of the aims of the Yemen FETP is to graduate highly competent multi-disciplinary public health professionals who can be positioned at influential posts in the public health structures especially on disease control and surveillance systems.

Since the Yemen FETP was established in 2011, five cohorts with 57 residents joined the Advance 2 year Program; about three quarters were from the governorates. By February 2020, 43 graduated; 25 (58%) are working at the central Ministry or governorate level covering disease control and surveillance system. The remaining graduates are working with partner organizations mainly the WHO country office and its governorates branches and involved in COVID-19 response. Furthermore, during 2017–2019 over 200 governorate and district surveillance frontline officers graduated from the 3 months (BFE)/ (PHEP), to improve disease surveillance and response (45). Additionally, the program organized six training workshops on outbreak investigations and Rapid Response during the same period. Through these workshops more than 100 surveillance officers and rapid response teams (RRTs) members were trained from different governorates, districts, and hospitals. Such trained surveillance officers and RRTs have been instrumental in the response to the COVID-19 epidemic. At the central level, four of the graduates (the Director General and the Deputy Director of the General Directorate for Disease Surveillance and Control, and the coordinator of Rapid Response Team and data manager at the Electronic Integrated Early Warning System (eIDEWS) are leading the COVID-19 preparedness and response activities at the central levels. Furthermore, the trained governorates and districts surveillance officers and RRTs are leading the COVID-19 response at the governorate and district levels.

Furthermore, since reporting of the first COVID-19 case from Yemen, the Yemen FETP organized four BFE/PHEP training sessions for surveillance officers from high-risk districts and organized refresher training for governorate surveillance officers on outbreak investigation. The Yemen FETP also conducted two special rapid response training sessions of COVID-19 for 50 participants from the governorates rapid response teams who are the key responders during public health emergencies. These training sessions provided the trainees with the core competencies needed to respond to public health threats of international concern especially the COVID-19.

Table 2 shows the role of Yemen FETP in building capacities of the health workforce to respond to COVID-19 outbreaks during 2020–2021.

**Table 2:** Yemen Field Epidemiology Training Program participation on building the health workforce capacity on COVID-19, Yemen, 2020-2021.

**Training**	**Target group**	**Duration**	**# of patches**	**# of participants**	**Supporting agency**
BFE/PHEP[Table-fn TN2]	District Surveillance Officers	3 months	4	100	WHO^*^, SFD^**^, EU^***^
RTT training for COVID-19[Table-fn TN3]	Governorate Team	5 days	3	75	WHO^*^, EMPHNET^****^
Outbreak investigation	Governorate Surveillance Officers	5 days	1	25	WHO^*^
IHR[Table-fn TN4]	Governorate Surveillance Officers	3 days	1	25	WHO^*^
IPC[Table-fn TN5]	Governorate hospital's team	2 days	6	150	WHO^*^, GAVI^*****^
Case management	Physicians at COVID-19 isolation centers	4 days	4	100	WHO^*^

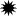
*Basic Field Epidemiology/Public Health Empowerment Program*.

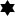
*Rapid Response team Training for COVID-19*.

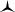
*International Health Regulations*.

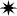
*Infection Prevention Control*.

* *World Health Organization*.

** *Social Fund for Development*.

*** *European Union*.

**** *Eastern Mediterranean Public Health Network*.

***** *Global Alliance for Vaccines and Immunization*.

Additionally, the Yemen FETP participated in the development of surveillance tools (e.g., case definition, contact tracing flow charts, and traveler screening forms) that were used by the residents and graduates who participated in the investigation of the first COVID-19 cases in Sana'a Capital.

### Pillar 4: Points of Entry (POE)

During 2017–2019, the Yemen FETP organized three training workshops on IHR. Through these workshops more than 50 POE officers working at national and governorates levels were trained on the key role they can play to prevent, prepare and respond to public health risks and prevent spread of the diseases through persons, conveyances and goods. These POE officers have played a crucial role in responding to COVID-19 pandemic.

Once the WHO declared COVID-19 as a PHEIC, the Yemen FETP graduates performed risk assessment at some POE using the WHO National capacities review tool for a nCoV to identify main gaps and plan for additional needs.

Furthermore, immediately after announcing the first case of COVID-19 in Yemen in April 2020, the Yemen FETP staff and graduates supported the special training organized by the eIDEWS for the governorate's surveillance and POE officers to increase their readiness for responding to COVID-19. Although the primary international airport of Yemen located in Sana'a is closed due to blockade and the only flights operating were the UN flights, Yemen FETP trained the Sana'a airport staff to identify and manage COVID-19 suspected cases.

Finally, the graduates and trained governorate and district surveillance officers helped screen the Yemeni returnees from China and other countries for potential COVID-19 symptoms. They also followed up those arrivals for appearance of early symptoms and referred suspected cases to the isolation centers.

### Pillar 5: National Laboratories

The Yemen FETP mandate is to support the One Health approach through training veterinarian and laboratory staff besides medical doctors. The program already enrolled 16 laboratory's staff in the advance Yemen FETP, three of them are nominated by the National Center of Public Health Laboratories (NCPHL), the designated Center for the COVID-19 diagnosis.

Moreover, the Yemen FETP graduates used the WHO National capacities review tool for a nCoV, to perform risk assessment at the NCPHL in order to identify main gaps and identify additional needs.

Nevertheless, the involvement of the Yemen FETP on national laboratories is still negligible and needs future strengthening.

### Pillar 6: Infection Prevention and Control

During 2017–2019, the Yemen FETP organized six training workshops on Biosafety and Biosecurity. During these workshops more than 100 Infection Prevention and Control (IPC) staff were trained from different tertiary and governorate public and private hospitals. Such capacity was crucial during the COVID-19 epidemic to ensure proper implementation of IPC measures at health facilities to prevent transmission to staff, patients, visitors and to the community.

Moreover, the Yemen FETP graduates used the WHO National capacities review tool for a nCoV, to perform risk assessments of IPC at some health facilities in order to identify gaps and plan for additional needs.

After the pandemic was announced, the Yemen FETP supported training for over 200 hospitals and health facilities personnel on IPC to prevent transmission to staff, patients, visitors and community.

### Pillar 7: Case Management

A senior Yemen FETP epidemiologist and two graduates developed the COVID-19 Standard Operating Procedures (SOPs) and Guidelines for Case Management. Furthermore, two Yemen FETP epidemiologists also participated in a special group from academicians, clinicians, and pharmacists to update the case management guidelines and assess the drug needs. The Yemen FETP Technical Advisor also participated in the COVID-19 Scientific Committee with other clinicians, academicians to support the development of treatment guidelines.

To ensure that staff are familiar with the suspected COVID-19 case definitions as well as able to deliver the appropriate care pathway and ensuring that patients with, or at risk of, severe illness are treated and/or referred immediately, the Yemen FETP supported training of 90 clinicians from hospitals that were designated for treating COVID-19 cases.

### Pillar 8: Operational Support and Logistics

Minimum role was played by the Yemen FETP on this pillar except for assessing the COVID-19 logistics, procurement and supply using the WHO National capacities review tool for a nCoV. Accordingly, Yemen FETP prepared a list of equipment and supplies needed for the response.

### Pillar 9: Maintaining Essential Health Services and Systems

The Yemen FETP advocated for ways to counteract the possible negative impact of COVID-19 on immunization and chronic diseases treatment. The program staff, graduates and residents participated in several webinar discussions on impact of COVID-19 and mitigation measures on immunization services and how to build resilient immunization programs that could reduce vaccination inequities and target public hesitancy and provider's reluctance. Furthermore, Yemen FETP encouraged responsible Immunization bodies (e.g., Expanded program on Immunization, Acute Flaccid Paralysis Surveillance, Measles Surveillance Program) and other public health specialists to participate in these discussions.

### Other Roles

Due to the initial lockdown and inability to continue in the in-class training sessions, Yemen FETP asked its residents to join online training sessions focused on COVID-19, using online platforms developed by different institutes/organizations such as John Hopkins University, University of Pittsburgh, St George's University, University of London, WHO Health Emergencies Program, and EMPHNET. Through using these platforms, the residents learned about a wide range of public health subjects and joined some specialized courses e.g., COVID-19 contact tracing, surveillance systems, epidemic/outbreaks, IPC etc. Regular circulars were also sent by the program to residents and graduates inviting them to participate in COVID-19 and related public health webinars organized by EMPHNET, Institute of Health Metrics and Evaluation, Arabic Public Health Association, and Yemeni Doctors in Diaspora.

Furthermore, the Yemen FETP participated in COVID-19 research (Table 3). Thus far, four articles have been published (18, 40, 41, 46) while more are under peer review or in preparation.

**Table 3:** Yemen field epidemiology training program participation on COVID-19 research.

**Authors**	**Title**	**Journal**	**Reference Number/N.B**
1. Al Nsour M, Bashier H, Al Serouri A, Malik E, Khader Y, Saeed K, Ikram A, Abdalla AM, Belalia A, Assarag B, Baig MA.	The role of the global health development/eastern mediterranean public health network and the eastern mediterranean field epidemiology training programs in preparedness for COVID-19.	JMIR public health and surveillance	(40)
2. Al Nsour M, Khader Y, Al Serouri A, Bashier H, Osman S.	Awareness and Preparedness of Field Epidemiology Training Program Graduates to Respond to COVID-19 in the Eastern Mediterranean Region: Cross-Sectional Study.	JMIR medical education	(41)
3. Al-Sakkaf E, Ghaleb Y, Al-Dabis E, Qairan M, Al-Amad M, Al-Serouri A, Al-Kohlani A.	First COVID-19 cases with high secondary infection among health workers, Sana'a capital, April (2020): Lessons learned and future opportunities.	International Journal of Infectious Diseases	(46)
4. Noman H, Dureab F, Ahmed I, Al Serouri A, Hussein T, Jahn A.	Mind the gap: an analysis of core capacities of the international health regulations (2005) to respond to outbreaks in Yemen.	BMC health services research	(18)
5. Ghaleb Y, Lami F, Al Nsour M, Rashak HA, Samy S, Khader YS, Al Serouri A, BahaaEldin H, Afifi S, Elfadul M, Ikram A.	Mental health impacts of COVID-19 on healthcare workers in the Eastern Mediterranean Region: a multi-country study.	Journal of Public Health	- Accepted - Funded by EMPHNET
6. Samy S, Lami F, Rashak HA, Al Nsour M, Eid A, Khader YS, Afifi S, Elfadul M, Ghaleb Y, Letaief H, Alaya NB.	Public health worker's knowledge, attitude and practice regarding COVID-19: the impact of Field Epidemiology Training Program in the Eastern Mediterranean Region.	Journal of Public Health (Oxford, England)	- Accepted - Funded by EMPHNET
7. Risk factors for COVID-19 Critical Outcomes in the Eastern Mediterranean Region EMRO region: A Multicounty Retrospective Study.			- Under review - Funded by the EMPHNET
8. Treatment Regimens used for Management of COVID-19 and their Effectiveness.			- Under review - Funded by the EMPHNET
9. Patient's Demographic, Clinical Characteristics and Outcomes and Risk Factors for Covid-19 Severity and Mortality in the Eastern Mediterranean Region: A Retrospective Study.			- Under review - Funded by the EMPHNET

## Conclusion

In Yemen, a country that is entering its seventh year of a protracted war, there are concerns regarding the quality of the COVID-19 reporting system especially as underreporting continues in some areas of the country due to a lack of testing facilities, delays in seeking treatment, stigma, difficulty accessing treatment centers, the perceived risks of seeking care or for political issues.

Despite all challenges, the Yemen FETP has been instrumental for the MoPHP during the COVID-19 epidemic preparedness and response. Through its dedicated staff, graduates, residents and trainees, the Yemen FETP has led the response through the WHO nine pillars of COVID-19 preparedness and response. Such efforts should be sustained by supporting the program to develop a strong cadre that is crucial for the Yemen public health system to respond to future epidemics.

Our results highlighted the need for health partners in Yemen and other developing and war-torn countries to continue working closely with health ministries to address COVID-19 especially in light of the emergence of new variants that are more infectious. These include assessing the COVID-19 impact on routine priority health programs; refining messaging to encourage behavioral change, and boosting ICU capacity. Moreover, health ministries and key partners should support the FETPs to continue their role in building and strengthening national and regional workforce capacity in field epidemiology as a strategy to improve disease surveillance and response and in the fight against the epidemic.

Furthermore, our study demonstrated for Yemen and other countries with similar context, especially during COVID-19 pandemic, that when access to outside experts becomes limited; it is crucial to invest in building local expertise to provide timely, cost-effective, and sustainable health services that meet the population's needs and are culturally appropriate. Those national experts are better equipped to respond to the health crisis as they know the country well, are well connected with the communities and can reach difficult or inaccessible areas. Furthermore, as the COVID-19 pandemic is still circulating with new variants that seem to be more infectious, it is essential to build such expertise at the governorate and district levels -as they are normally the first respondents- and to provide them with the necessary tools for immediate response in order to overcome the disastrous delays.

Finally, this study provides an overview of the FETP in Yemen and its efforts to ensure the timely COVID-19 detection and response and save lives; however, it has some limitations. First, as the decision in the North not to publicly report COVID-19 cases, presumably to prevent panic and overwhelm the public health system, the role of the FETP in responding to COVID-19 was not well-apparent, depriving it of much-needed visibility for future funding support and recruitment of residents. Second, since this manuscript was authored by the Yemen FETP team, the findings regarding the program's performance in responding to COVID-19 should be considered internal rather than a full-fledged evaluation performed by external evaluators.

## Author Contributions

All authors contributed to drafting, revising, and approving the final manuscript, had access to all the data, and agree to be accountable for all aspects of the work.

## Conflict of Interest

The authors declare that the research was conducted in the absence of any commercial or financial relationships that could be construed as a potential conflict of interest.

## Publisher's Note

All claims expressed in this article are solely those of the authors and do not necessarily represent those of their affiliated organizations, or those of the publisher, the editors and the reviewers. Any product that may be evaluated in this article, or claim that may be made by its manufacturer, is not guaranteed or endorsed by the publisher.
